# Molecular Prevalence of Equine Parvovirus-Hepatitis in the Sera of Clinically Healthy Horses in South Korea

**DOI:** 10.3390/vetsci8110282

**Published:** 2021-11-19

**Authors:** Sang-Kyu Lee, Dongsun Park, Inhyung Lee

**Affiliations:** 1 Veterinary Centre, Korea Racing Authority, Gwacheon 13822, Korea; bestvet@kra.co.kr; 2 Department of Biology Education, Korea National University of Education, Cheongju 28173, Korea; dvmdpark@knue.ac.kr; 3 College of Veterinary Medicine, Seoul National University, Seoul 08826, Korea

**Keywords:** EqPV-H, prevalence, molecular prevalence, phylogenetic analysis, South Korea

## Abstract

Equine parvovirus-hepatitis (EqPV-H) causes equine hepatitis. The prevalence of EqPV-H in healthy horses has been reported in the United States, China, Germany, and Austria. The present study determined the prevalence of EqPV-H in the sera of clinically healthy horses in South Korea to identify the potential factors for infection and examine the genetic diversity of EqPV-H DNA sequences through comparison with foreign strains. Serum samples collected from 321 horses were tested for EqPV-H using non-structural protein 1 (NS1)-specific polymerase chain reaction. The associations of EqPV-H infection with sex, age, aspartate aminotransferase and γ-glutamyl transferase levels, and race performance were analyzed. Fourteen samples tested positive for EqPV-H (4.4%, 14/321), and EqPV-H infection was associated with sex (*p* = 0.006) and performance (*p* = 0.049). In both EqPV-H-positive and control horses, liver-specific biochemical analytes were within the normal ranges. Phylogenetic analyses based on the partial sequences of EqPV-H NS1 revealed that the Korean EqPV-H isolates shared approximately 98.7–100% similarity. Of these, 11 Korean isolates shared high similarity with strains from the United States, Germany, and China, and the remaining three strains were distinct in phylogenetic analyses. The present study describes the current molecular prevalence, potential risk factors, and genetic diversity of Korean EqPV-H.

## 1. Introduction

Equine parvovirus-hepatitis (EqPV-H; *Ungulate copiparvovirus 6*) is a single-stranded DNA virus of the genus *Copiparvovirus* belonging to the family Parvoviridae [1,2,3]. EqPV-H genome size is 5308 nt, and it encodes two open reading frames and non-structural (non-structural protein 1; NS1) and capsid (viral protein; VP) proteins [1,4,5]. In 2018, Divers et al. reported EqPV-H for the first time in a case of Theiler’s disease (i.e., equine serum hepatitis or idiopathic acute hepatic disease) [6] in the United States [1]. Theiler’s disease is the most frequent cause of acute and probably fatal viral equine hepatitis [7,8,9]. This disease has been linked to EqPV-H infection, with or without the previous administration of equine biological products [10,11,12,13]. It mostly occurs 4–10 weeks after the administration of a biological substance of equine origin, including tetanus and botulinum antitoxin or equine serum and plasma [1,6,13,14,15,16], with the incidence rate of 1.4–18% [1,6,12].

Since its first report in 2018, recent studies in the United States and Slovenia have speculated the association of EqPV-H with Theiler’s disease [12,13,17]. High positivity rates of EqPV-H have been reported in cases of Theiler’s disease, ranging 90% to 100% in the United States [12,13] and 100% in Slovenia [17]. Moreover, EqPV-H DNA was detected in commercial equine serum pools originating from the United States, Canada, New Zealand, Italy, and Germany [4], with positivity rates as high as 61.1% [4]. EqPV-H is an important pathogen associated with Theiler’s disease, and, in addition to clinical cases, subclinical EqPV-H infections have been documented [10]. The prevalence of EqPV-H in healthy horses has been investigated in only four countries, although the viral DNA was less frequently detected in healthy horses (7.1–13%) [1,9,18,19]. Furthermore, the positivity rate of EqPV-H DNA in the sera of healthy horses was 13.0% in the United States [1], 8.3–11.9% in China [19,20], 7.1% in Germany [9], and 8.9% in Austria [18]. The molecular detection and phylogenetic analysis of EqPV-H are commonly performed using NS1-specific polymerase chain reaction (PCR) [1,4,11,19], and previous studies have reported low genetic diversity amongst EqPV-H isolates [1,4,11,19,20,21].

Given its recent discovery, little is known regarding the geographic distribution, epidemiology, and genetic diversity of EqPV-H. According to the importance and potential harmfulness of EqPV-H as a causal agent of viral hepatitis in horses [10,22], investigation of its prevalence in South Korea is urgently needed. Therefore, the present study determined the incidence of EqPV-H in the sera of clinically healthy horses at Korea Racing Authority (KRA) properties in South Korea using molecular methods to identify the potential risk factors for infection and explore the genetic relationships of Korean EqPV-H with foreign isolates based on the NS1 sequences.

## 2. Materials and Methods

### 2.1. Sample Size Calculation and Collection

Considering the approximate population of 35,606 horses in South Korea [23], the appropriate sample size was calculated. The expected prevalence of EqPV-H DNA in horses was 9.8% based on the average prevalence of EqPV-H DNA in the sera of healthy horses reported in previous studies [1,10,11,12,13]. Sample size was calculated with Ausvet’s Epitools [24]. The KRA conducts a regular equine infectious disease surveillance program with approximately 10% samples of the KRA horse population to protect against an outbreak of equine infectious diseases at the KRA. Considering the confidence level of 95% and accepted absolute error of 5%, at least 136 horses are required. In the present study, samples from 321 clinically healthy horses on four properties of the KRA in four provinces in South Korea were collected in 2021. The average age of the horses was 5.9 (2–23) years. Specifically, 153 samples were collected from horses at KRA Seoul in Gyeonggi province, 30 from horses at KRA Jangsu in Jeonbuk province, 80 from horses at KRA Busan in Gyeongnam province, and 58 from horses at KRA Jeju in Jeju province. All horses were subjected to routine clinical examinations, and no clinical signs of suspected hepatitis, including anorexia, icterus, and ataxia, were observed. Immediately after collection, the samples were stored at −80 °C until use. Data on sex, age, breed, purpose, and property were recorded.

### 2.2. Sample Processing and PCR

DNA was extracted from the serum samples using the Maxwell^®^ RSC Viral Total Nucleic Acid Purification Kit (Promega, Madison, WI, USA) on the Maxwell^®^ RSC 48 Instrument (Promega) according to the manufacturer’s instructions and stored at –70 °C until use.

For EqPV-H DNA screening, the EcPV-NS1 primers EqPV-H ak1 (5′-GGAGAAGAGCGCAACAAATGCA-3′) and EqPV-H ak2 (5′-AAGACATTTCCGGCCGTGAC-3′) were used in the first round of nested PCR and the primers EqPV ak3 (5′-GCGCAACAAATGCAGCGGTTCGA-3′) and EqPV-H ak4 (5′-GGCCGTGACGACGGTGATATC-3′) were used in the second round of nested PCR [1]. In the nested PCR round, a 435 bp partial fragment of the NS1 gene was amplified [1]. PCR was performed with the HotStarTaq Plus Master Mix Kit (Qiagen, Hilden, Germany) in a 20 µL reaction system containing HotStarTaq Plus DNA Polymerase (Qiagen) (1 U), 1 μL of primers (10 μM), dNTP mixture (400 μM), a reaction buffer with 3 mM MgCl_2_, and 2.5 µL of template DNA. The reaction conditions were as follows: 5 min of denaturation at 95 °C, followed by 35 cycles of 30 s at 94 °C, 30 s at 60 °C, and 1 min at 72 °C, and final extension of 10 min at 72 °C. PCR was performed on the SimpliAmp Thermal Cycler (Applied Biosystems Inc., Waltham, MA, USA). The serum samples (accession number: OK336512) for EqPV-H stored at the KRA Veterinary Centre, which were confirmed through DNA sequence analysis, were used as the positive controls, and nuclease-free water (Qiagen) was used as the negative control. The PCR products were visualised on 1.5% Tris–borate–EDTA (TBE) agarose gels using Safe Shine Green (Biosesang, Seongnam-si, South Korea) under UV light.

The second PCR was performed on positive samples using the first EqPV-H NS PCR production. In the second round, a 587 bp partial fragment of the NS1 region was amplified using the primers EPVseqr01 (5′-TGGTTGGTGACGCCTGTC-3′) and 1104R (5′-GGGAATGTCATTGAACGGGAA-3′) [4]. PCR was performed with the HotStarTaq Plus Master Mix Kit (Qiagen) in a 50 µL reaction system containing 2 µL of extracted DNA and 2.5 μM of each primer. The PCR conditions were as follows: 5 min denaturation at 95 °C, followed by 35 cycles of 30 s at 94 °C, 30 s at 60 °C, and 1 min at 72 °C, and final extension of 10 min at 72 °C. The amplicons were detected using electrophoresis as described above, purified using the PureLink™ Quick Gel Extraction and PCR Purification Combo Kit (Invitrogen™, Waltham, MA, USA), and sequenced using the ABI PRISM BigDye Terminator Cycle Sequencing Ready Reaction Kit V.3.1, with the ABI3730 Genetic Analyzer (Applied Biosystems). After sequencing the amplicons with the same primers, the results were merged and homology of the deduced nucleotide sequences for EqPV-H was analyzed with the GenBank database (http://www.ncbi.nlm.nih.gov/Genbank/, accessed on 27 September 2021) using BLAST (http://blast.ncbi.nlm.nih.gov/Blast.cgi, accessed on 27 September 2021).

### 2.3. Phylogenetic Analysis

In phylogenetic analyses, the genetic relationships of the obtained Korean EqPV-H with isolates from other countries were determined. Thirty-seven nucleotide sequences of the EqPV-H NS1 gene were retrieved from GenBank and included in the analysis. Sequences of the EqPV-H NS1 gene identified in the present study were aligned with those retrieved from the GenBank in BioEdit v.7.2.6 (BioEdit, RRID:SCR_007361, Los Altos, CA, USA) and MEGA X [25] using the Clustal W algorithm. Sequences were trimmed to the partial NS1 sequence length of the PCR product. Phylogenetic trees were constructed using the maximum likelihood method with 1000 bootstrap values using MEGA X [25], and a general time-reversible model was applied [26].

### 2.4. Serum Biochemistry

Serum samples of all EqPV-H DNA-positive horses were tested for biochemical analytes indicative of hepatic disease. As control horses, double-negative samples for EqPV-H were submitted to biochemical assays. As biochemical parameters, aminotransferase (AST) and γ-glutamyl transferase (GGT) levels were tested using Dri-Chem 3500i (Fujifilm, Tokyo, Japan). The reference ranges used in the present study were as follows: 226–336 U/L for AST and 4–44 U/L for GGT [27]. Values exceeding the upper limit of the reference range were regarded as elevated.

### 2.5. Statistical Analysis

For statistical analysis based on sex, the horses were grouped as males (*n* = 219; 109 stallions and 110 geldings) and females (*n* = 102). For statistical analysis based on age, the horses were divided into three groups: 2–4 years old (*n* = 142), 5–7 years old (*n* = 120), and >8 years old (*n* = 59). The chi-square test was used to analyze the differences in the frequency distribution of horses with and without EqPV-H DNA positivity amongst different sexes and age groups. The odds ratio (OR), confidence interval (CI) of the OR, and *p*-value were evaluated for the pairwise comparison of horses with and without EqPV-H DNA positivity between different sexes and different age groups. For statistical analysis based on the performance level of thoroughbred racehorses, the animals were divided into three groups based on the results of a race conducted on the sample collection day. The performance level was calculated as the percentage of rank divided by the number of participating animals in the race: high (≤1/3 (3.3%)), moderate (>1/3 (33.3%) and ≤2/3 (66.7%)), and low (>2/3 (66.7%)). The proportion of the performance levels was compared according to EqPV-H infection. To confirm the effect of EqPV-H infection on the performance level, the proportion of performance levels was compared between the EqPV-H-positive and EqPV-H-negative groups using Mantel–Haenszel chi-square test (linear-by-linear association). To minimise the effects of variants in race results, the race results of EqPV-H-negative horses were recruited from the horses that trained by the same trainers and that participated in races on the same race day. Statistical significance was set at *p* ≤ 0.05, and all analyses were performed using IBM SPSS Statistics v22 (IBM Corp., Armonk, NY, USA).

## 3. Results

### 3.1. Prevalence of EqPV-H DNA in the Sera of Clinically Normal Horses

A total of 321 serum samples was collected from horses reared on four properties of the KRA in four provinces to investigate the prevalence of EqPV-H DNA in clinically healthy horses, mainly thoroughbred racehorses, at the KRA in South Korea. EqPV-H DNA was detected in 14 of the 321 horse serum samples (4.4%) using NS1-speficic PCR (Table 1). Based on the detection results, the positive rate of EqPV-H in the sera of clinically healthy horses was lower in South Korea than in other countries [1,10,11,12,13]. The proportion of the positive rate of EqPV-H was investigated based on horse breed, property, sex, purpose, and age (Table 1). The positive rate was comparable between the stallions and geldings (6.4% and 6.4%, respectively), but none of the mares was positive (0%). Likewise, the positive rate was comparable amongst thoroughbreds (4.6%), pony (3.1%), and other breeds (3.3%). According to the purpose, the positive rate amongst riding horses (12.0%) was higher than that amongst race (4.3%), breeding (0%), or other horses (0%). Furthermore, according to the property, the samples collected from Busan (5/80, 6.3%) and Seoul (9/153, 5.9%) were positive, whereas those collected from Jangsu (0/30, 0%) and Jeju (0/58, 0%) were negative. However, samples were biased to thoroughbred racing horses in this study. Since the Korea Racing Authority is a horse-racing company, samples included in this study were mainly from thoroughbred racing horses, especially in KRA Seoul and Busan. Therefore, similar sample sizes per group based on factors, including property, purpose, and breed, were limited in this study. The relationship between EqPV-H infection and these factors could not be concluded, because of this limitation. The higher positive rate of EqPH-V amongst riding horses from the property in Busan may be attributed to the relatively limited sample size per group based on purpose and property in the present study. Detailed information regarding the serum samples is presented in Table 1.

### 3.2. Phylogenetic Analysis

**Figure 1:**
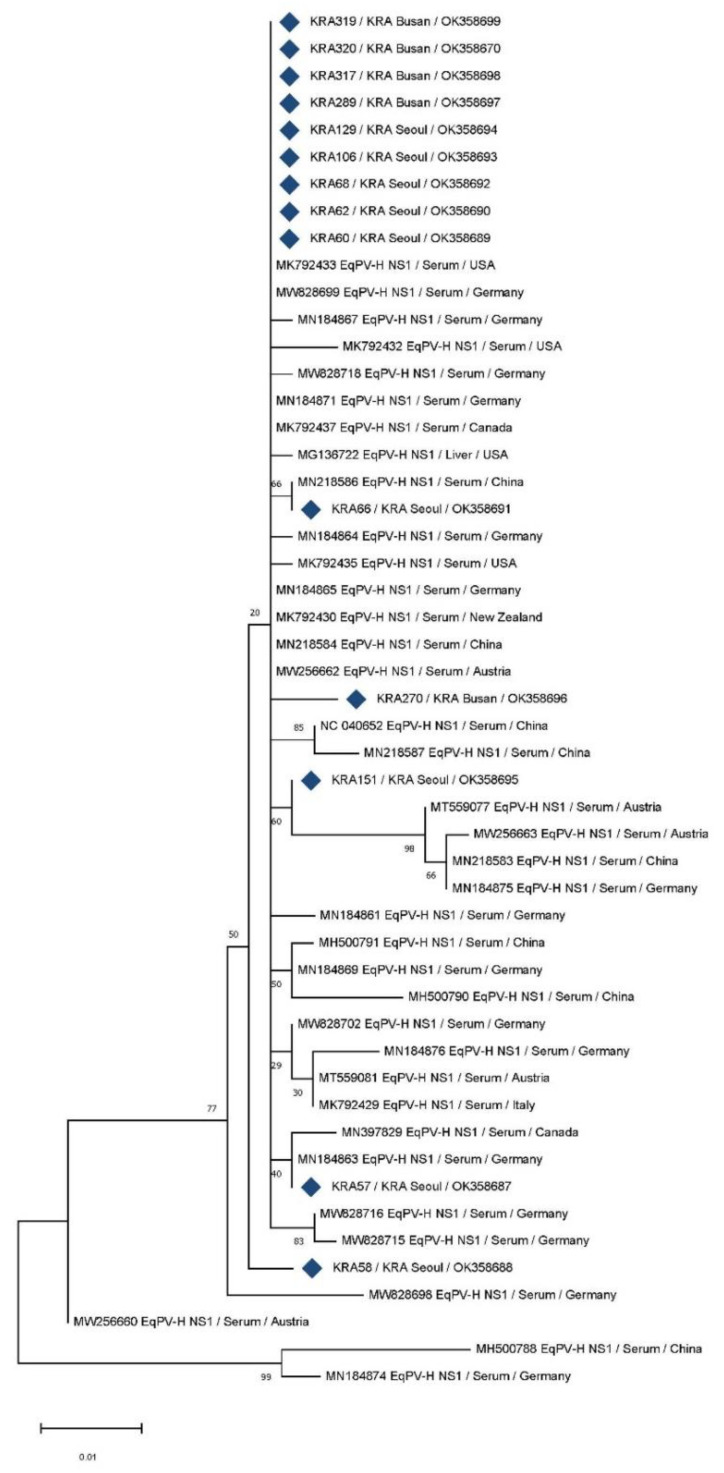
Phylogenetic tree constructed using the maximum likelihood method based on the partial NS1 gene sequences of equine parvovirus-hepatitis (EqPV-H). All positions containing gaps and missing data were eliminated, resulting in 475 positions in the final set. Sequences of Korean horses are indicated with blue diamonds, with sample number, property, and GenBank accession numbers in parentheses. For foreign EqPV-H strains, GenBank accession numbers, isolation source, and country are shown in parentheses. Bootstrap values are shown on the branches.

Partial sequences of the NS1 gene of 14 EqPV-H strains isolated from Korean horses were compared with those of 37 foreign strains retrieved from GenBank, harbouring 475 positions, to explore the genetic diversity of EqPV-H strains in South Korea (Figure 1). The foreign strains were isolated from seven countries, namely the United States, Germany, Canada, China, New Zealand, Austria, and Italy. The partial nucleotide sequences of the NS1 gene of the 14 Korean EqPV-H strains shared approximately 98.7–100% similarly amongst one another and 93.1–100% similarity with the foreign strains.

The sequences of nine Korean isolates (KRA60, 62, 68, 106, 129, 289, 317, 319, and 320) in the present study were clustered with the retrieved sequences of isolates from the United States and Germany. The Korean isolate KRA57 was clustered with an isolate from Germany, and the Korean isolate KRA66 was clustered with an isolate from China. The Korean isolate KRA151 formed a separate branch close to the isolates from Austria, China, and Germany. The Korean strains KRA58 and KRA270 each formed a separate branch.

### 3.3. Liver-Specific Biochemistry

**Figure 2:**
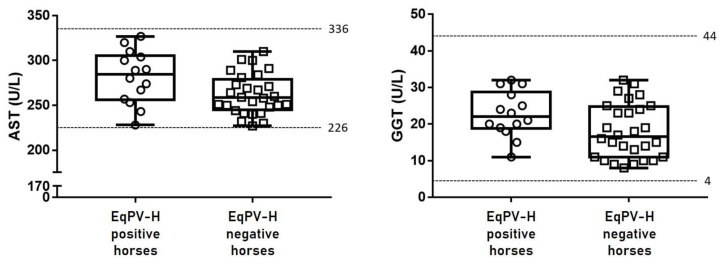
Liver-specific biochemical parameters (aspartate aminotransferase (AST; reference range: 226–336 U/L) and γ-glutamyl transferase (GGT; reference range: 4–44 U/L)) in the sera of both equine parvovirus-hepatitis (EqPV-H)-positive (*n* = 14) and EqPV-H-negative (control) horses (*n* = 28). Values of both EqPV-H-positive and control horses were within the normal ranges, indicating that the animals were clinically healthy.

Fourteen EqPV-H-positive and 28 EqPV-H-negative serum samples (controls) were tested for two liver-associated biochemical parameters (AST and GGT). All samples were clinically healthy, and the values of biochemical analytes in the sera of both 14 EqPV-H-positive and 28 EqPV-H-negative horses were within the normal ranges (Figure 2).

### 3.4. EqPV-H Infection According to Sex, Age, and Performance

The prevalence of EqPV-H infection according to sex, age, and performance was tested. The chi-square test revealed statistically significant differences in the frequency distribution of horses with and without EqPV-H DNA between males and females (χ^2^_(1)_ = 6.797; *p* = 0.006); however, there were no significant differences amongst the age groups (χ^2^_(2)_ = 0.247; *p* = 0.939). Subsequent pairwise analyses revealed that EqPV-H positivity was not significant in the age groups (*p* = 0.765, *p* > 0.99 1, and *p* > 0.99) (Table 2). The proportion of performance levels was linearly higher in the EqPV-H-negative group but linearly lower in the EqPV-H-positive group (Table 3 and Table S1). The Mantel–Haenszel chi-square test (linear-by-linear association) confirmed the effect of EqPV-H infection on performance level. EqPV-H infection could increase or decrease the performance level of thoroughbred racehorses (*p* = 0.049). EqPV-H infection tended to reduce the performance level of racehorses (Table 3). Overall, male horses are at a greater risk of EqPV-H infection, and EqPV-H may impede the performance of racehorses.

## 4. Discussion

As a major causative viral agent of Theiler’s disease [28], EqPV-H has recently garnered much attention, and since its first report in the United States in 2018, it has been detected in Germany, Austria, Slovenia, and China [1,9,17,18,19,20]. EqPV-H has been detected in cases of Theiler’s disease and is also prevalent in clinically healthy horses [10,22]. In the present study, the prevalence of EqPV-H in the serum of clinically healthy horses, mainly thoroughbred racehorses, at the KRA in South Korea was determined, along with the evaluation of the potential risk factors for its infection and phylogenetic analysis of Korean and foreign viral isolates. As such, the prevalence of EqPV-H in South Korean horses was relatively low (4.4%, 14/321).

The prevalence of EqPV-H in horse populations can vary according to the geographic location, age, breeding history, and sampling season [9,18,22,28]. In the present study, the positive rate of EqPV-H (4.4%) in South Korea (Table 1) was lower than that reported in previous studies (7.1–13%) [1,9,18,19,20]. In previous studies, the samples were collected from horses of mixed breeds [18,19,20] or groups of similar age distribution [9,18]. In the present study, since all samples were collected from horses reared on the properties of the KRA, a horse racing company, most included animals were thoroughbred (80.7%, 259/321) and bred for the purpose of racing (88.0%, 228/259). Thoroughbred racehorses seem to be managed more carefully because of their relatively high economic value and relatively younger horses are used for the athletic ability. Thus, being bred for racing, the health status of the included horses was carefully managed and they were relatively young (average 4.4 years old). Overall, the proportion of healthy thoroughbred race horses of the total sample included in the present study (71.0%, 228/321) was higher than that in two previous studies during 2018 and 2020 in China (45.5%, 65/143 and 41.7%, 25/60, respectively), [19] and another study during 2019 in Germany (0%, 0/392) [9]. In previous studies by Divers et al. [1] and Badenhorst et al. [18], the proportions of specific breeds in the sampled horses were not described. In addition, administration of equine-origin biological products is a proven transmission route of EqPV-H infection [10,22,28,29]. However, the use of equine-originated products seems to be infrequent in equine veterinary practice in South Korea because no equine-origin biological products have yet been registered and approved in South Korea [30]. Since EqPV-H can be transmitted via blood products, its infection via vector mechanical transmission during the spring and summer has been suggested considering the incubation period [12]. However, in the present study, the samples were collected from April to June. Taken together, it is thought that the majority of thoroughbred racehorses in sampled horses, limited usage of equine-origin biological products in South Korea and the different sampling season may explain the lower positivity of EqPV-H in Korean horses in the present study.

In a study in Austria, sex was not associated with EqPV-H infection [18]; however, in the present study, sex was significantly associated with EqPV-H infection (*p* = 0.009). Specifically, 7/109 stallions (6.4%) and 7/110 geldings (6.4%) were EqPV-H-positive, whereas 0/102 mares were EqPV-H-negative (0%). Therefore, in South Korea, male horses may be at a higher risk of EqPV-H infection than female horses. However, the reason for the high positive rate of EqPV-H infection in male horses remains unclear, and the correlation between sex and EqPV-H infection warranted further study.

Previous studies have reported advanced age as a risk factor for EqPV-H infection in clinically healthy horses [9,18]. In the present study, however, there was no correlation between age and EqPV-H infection in Korean horses (Table 2). As such, EqPV-H positivity was similar across the three age groups, ranging from 3.4% to 5.0%, and the EqPV-H infection was not statistically significant. This trend may be attributed to the inclusion of more young horses (2–6 years old: 248/321, 77.3%) relative to older horses (7–23 years old: 73/321, 22.7%).

EqPV-H was detected in KRA Seoul and KRA Busan properties, but not in KRA Jangsu and KRA Jeju properties. Therefore, EqPH-V presumably circulates amongst these properties, and regular EqPV-H screening would be beneficial to evaluate the risk of EqPV-H outbreak on these properties. The EqPV-H positivity rate was the highest in riding horses (12%, 3/25), followed by racehorses (4.3%, 11/257), and the virus was not detected in breeding (0%, 0/16) and other horses (0%, 0/23). Thoroughbred horses showed highest EqPV-H-positive rate (4.6%, 12/259), followed by other breeds (3.3%, 1/30) and pony (3.1%, 1/32). However, given the limitation of the difference in sample size across groups in the present study, a conclusion on the relevance of EqPV-H positivity with property, purpose, and breed could not be drawn. Further studies are required to determine the association of property, purpose, and breed among similar size of groups per factor with EqPV-H infection in South Korea.

Although EqPV-H-infected horses were clinically healthy [1,18,19,20], the potentially detrimental effects of this infection on the athletic performance have been speculated. In the present study, serum samples were collected from thoroughbred racehorses on the day of race, and the effects of EqPV-H on race results were analyzed. The performance levels of the majority of EqPV-H-positive horses was low (5/11, 45.5%), but that of EqPV-H-negative horses was high (7/11, 63.6%) (Table 3). The Mantel–Haenszel chi-square test (linear-by-linear association) revealed that EqPV-H infection and performance level were significantly associated in thoroughbred racehorses (*p* = 0.049).

Partial sequences of the NS1 gene of EqPV-H strains were used to establish the phylogenetic relationships amongst Korean isolates and foreign strains from GenBank (Figure 1). The South Korean EqPV-H strains showed low genetic diversity (98.7–100% similarity) amongst themselves but slightly greater diversity compared with the foreign strains (93.1–100% similarity). The EqPV-H NS1 gene is highly conserved, with low genetic diversity [1,4,9,11,19,20]. Consistent with previous reports based on the serum samples of clinically healthy horses in the United States in 2018 (<2%) [1], China in 2018 (<3%) [19], China in 2020 (<3%), and Austria in 2021 (<5%) [18], this study noted low genetic diversity of EqPV-H NS1 sequences in South Korean samples (<2%). Phylogenetic analysis based on the NS1 sequences of Korean and foreign strains showed that most sequences originating from Korean horses (9/11) were clustered with previously published foreign sequences. Five isolates from the KRA Seoul property (KRA 60, 62, 68, 106, and 129) and four isolates from the KRA Busan property (KRA289, 317, 319, and 320) were clustered with strains from the United States and Germany. Two isolates from the KRA Seoul property (KRA57 and 66) were clustered with the German and Chinese strains, respectively. Two other isolates from the KRA Seoul property (KRA 58 and 151) and one isolate from the KRA Busan property (KRA270) were distinct from the previously reported strains and formed separate branches. Based on these results, genetically similar EqPV-H strains are primarily circulating in the KRA Seoul and Busan properties (nucleotide sequence similarity, 98.7–100%), with few distinct strains. The EqPV-H NS1 sequences of Korean isolates shared high similarity with previously published sequences from the United States, Germany, and China.

Interestingly, the EqPH-V positivity rate of riding horses was high (12%), and all riding horses that tested positive for EqPH-V (*n* = 3) were reared in the same stable at the KRA Busan property; moreover, these isolates (KRA317, 319, and 320) formed a cluster in phylogenetic analysis, with 100% nucleotide sequence homology. EqPV-H-infected horses can spread the virus via nasal, oral, and fecal routes for over 10 weeks, and the oral route of infection has been recently proven [31]. However, there was no clinical history of blood product administration in the three EqPH-V-positive horses from KRA Busan. Considering that the infected horses were reared in the same stable and the 100% similarity of their NS1 sequences, the EqPV-H infection in these animals likely occurred through non-iatrogenic horizontal transmission. However, the other EqPV-H-positive horses (*n* = 11) had stayed at each different stable, thus the possibility of EqPV-H infection from other horses could not be investigated in this study.

To investigate whether EqPV-H infection affected the liver, AST and GGT levels were evaluated. However, these values were within the normal ranges in both EqPH-V-positive and EqPH-V-negative horses (Figure 2). These results are consistent with previous reports in clinically healthy horses that the serum levels of liver-associated biochemical parameters were within their normal ranges even in EqPH-V-positive horses (0/13 in the United States [10], 0/17 in China [19], and 0/23 in Austria [18]). Although EqPV-H infection can cause acute and fatal hepatitis in horses, most infected animals appear clinically healthy [10,13,22]. Therefore, further studies are warranted to determine the reasons for differences in the occurrence of clinical hepatitis amongst EqPV-H-infected horses.

## 5. Conclusions

The present study illustrated the prevalence of EqPV-H in clinically healthy horses, mainly thoroughbred racehorses, at the KRA in South Korea. In the tested horses, the virus positivity rate was low, and the identified EqPV-H strains exhibited low genetic diversity. EqPV-H infection was associated with sex (male (6.4%) vs. female (0%), *p* = 0.006) in South Korean horses and reduced performance level (*p* = 0.049) in thoroughbred racehorses. Further investigation is warranted to elucidate the reasons for differences in clinical hepatic pathogenicity in EqPV-H-infected horses.

## Figures and Tables

**Table 1:** Detailed information of property location, number, breed, purpose, and age of equine samples tested for equine parvovirus-hepatitis (EqPV-H) in South Korea.

Property	Number (Sample ID)	Breed			Sex			Purpose				EqPV-H-Positive	Age (Years)		
		TB	P	O	S	G	M	RC	RD	B	O		2–4	5–7	≥8
KRA Seoul	153 (KRA1-153)	9/153	0/0	0/0	7/66	2/44	0/43	9/153	0/0	0/0	0/0	9/153 (5.9%)	4/74	5/78	0/1
KRA Busan	80 (KRA242-321)	3/76	1/2	1/2	0/26	5/20	0/34	2/75	3/5	0	0	5/80 (6.3%)	2/59	1/16	2/5
KRA Jangsu	30 (KRA154-183)	0/16	0/2	0/12	0/13	0/6	0/11	0/0	0/0	0/16	0/14	0/30 (0%)	0/3	0/9	0/18
KRA Jeju	58 (KRA184-241)	0/14	0/28	0/16	0/4	0/40	0/14	0/29	0/20	0/0	0/9	0/58 (0%)	0/6	0/17	0/35
Total	321	12/259	1/32	1/30	7/109	7/110	0/102	11/257	3/25	0/16	0/23	14/321 (4.4%)	6/142	6/120	2/59

Abbreviations: TB, thoroughbred; P, pony; O, others; S, stallion; G, gelding; M, mare; RC, racing; RD, riding; B, breeding; KRA, Korea Racing Authority.

**Table 2:** Odds ratio (OR), confidence interval (CI) of OR, and *p*-value for the pairwise comparison of horses with and without equine parvovirus-hepatitis (EqPV-H) infection according to sex and age. For statistical analysis based on sex, stallions and geldings were grouped as male and EqPV-H-negative horses were compared with EqPV-H-positive horses. For statistical analysis based on age, EqPV-H-negative horses were compared with EqPV-H-positive horses.

Comparison	*p*-Value	OR	95% CI of OR	
			Lower Limit	Upper Limit
Sex				
Male vs. Female	0.006	0.936	0.904	0.969
Age				
2–4-year-old vs. 5–7-year-old	0.765	0.838	0.263	2.67
2–4-year-old vs. >8-year-old	>0.99	1.257	0.246	6.417
5–7-year-old vs. >8-year-old	>0.99	1.5	0.293	7.668

Abbreviations: OR, Odds ratio; CI, confidence interval.

**Table 3:** Performance levels of equine parvovirus-hepatitis (EqPV-H)-positive and EqPV-H-negative thoroughbred racehorses in South Korea (*n* = 22).

EqPV-H Status	Number	Performance Levels		
		High (≤33.3%)	Moderate (>33.3% to ≤66.7%)	Low (>66.7%)
Positive	11	2 (18.2%)	4 (36.4%)	5 (45.5%)
Negative	11	7 (63.6%)	2 (18.2%)	2 (18.2%)

## Data Availability

Sequences generated in this study were submitted to GenBank under the accession numbers from OK358687 to OK358700.

## Supplementary Materials

The following are available online at https://www.mdpi.com/article/10.3390/vetsci8110282/s1, Table S1: Detailed race results of thoroughbred racehorses used to analyze the association between performance level and equine parvovirus-hepatitis (EqPV-H) infection.

## References

[1] Divers T.J., Tennant B.C., Kumar A., McDonough S., Cullen J., Bhuva N., Jain K., Chauhan L.S., Scheel T.K.H., Lipkin W.I. (2018). New Parvovirus Associated with Serum Hepatitis in Horses after Inoculation of Common Biological Product. Emerg. Infect. Dis..

[2] Pénzes J.J., Söderlund-Venermo M., Canuti M., Eis-Hübinger A.M., Hughes J., Cotmore S.F., Harrach B. (2020). Reorganizing the family Parvoviridae: A revised taxonomy independent of the canonical approach based on host association. Arch. Virol..

[3] Walker P.J., Siddell S.G., Lefkowitz E.J., Mushegian A.R., Adriaenssens E.M., Dempsey D.M., Dutilh B.E., Harrach B., Harrison R.L., Hendrickson R.C. (2020). Changes to virus taxonomy and the Statutes ratified by the International Committee on Taxonomy of Viruses (2020). Arch. Virol..

[4] Meister T.L., Tegtmeyer B., Postel A., Cavalleri J.V., Todt D., Stang A., Steinmann E. (2019). Equine Parvovirus-Hepatitis Frequently Detectable in Commercial Equine Serum Pools. Viruses.

[5] Cotmore S.F., Agbandje-McKenna M., Chiorini J.A., Mukha D.V., Pintel D.J., Qiu J., Soderlund-Venermo M., Tattersall P., Tijssen P., Gatherer D. (2014). The family Parvoviridae. Arch. Virol..

[6] Theiler A. (1918). Acute liver-atrophy and parenchymatous hepatitis in horses. 5th and 6th Repts. of the Director of Veterinary Research.

[7] Reinecke B., Klöhn M., Brüggemann Y., Kinast V., Todt D., Stang A., Badenhorst M., Koeppel K., Guthrie A., Groner U. (2021). Clinical Course of Infection and Cross-Species Detection of Equine Parvovirus-Hepatitis. Viruses.

[8] Sturgeon B. (2017). Theiler’s disease. Vet. Rec..

[9] Meister T.L., Tegtmeyer B., Brüggemann Y., Sieme H., Feige K., Todt D., Stang A., Cavalleri J.V., Steinmann E. (2019). Characterization of Equine Parvovirus in Thoroughbred Breeding Horses from Germany. Viruses.

[10] Divers T.J., Tomlinson J.E. (2020). Theiler’s disease. Equine Vet. Educ..

[11] Baird J., Tegtmeyer B., Arroyo L., Stang A., Brüggemann Y., Hazlett M., Steinmann E. (2020). The association of Equine Parvovirus-Hepatitis (EqPV-H) with cases of non-biologic-associated Theiler’s disease on a farm in Ontario, Canada. Vet. Microbiol..

[12] Tomlinson J.E., Tennant B.C., Struzyna A., Mrad D., Browne N., Whelchel D., Johnson P.J., Jamieson C., Löhr C.V., Bildfell R. (2019). Viral testing of 10 cases of Theiler’s disease and 37 in-contact horses in the absence of equine biologic product administration: A prospective study (2014–2018). J. Vet. Intern. Med..

[13] Tomlinson J.E., Kapoor A., Kumar A., Tennant B.C., Laverack M.A., Beard L., Delph K., Davis E., Schott Ii H., Lascola K. (2019). Viral testing of 18 consecutive cases of equine serum hepatitis: A prospective study (2014–2018). J. Vet. Intern. Med..

[14] Marsh H. (1937). Losses of undetermined cause following an outbreak of equine encephalomyelitis. J. Am. Vet. Med. Assoc..

[15] Thomsett L.R. (1971). Acute hepatic failure in the horse. Equine Vet. J..

[16] Chandriani S., Skewes-Cox P., Zhong W., Ganem D.E., Divers T.J., Van Blaricum A.J., Tennant B.C., Kistler A.L. (2013). Identification of a previously undescribed divergent virus from the Flaviviridae family in an outbreak of equine serum hepatitis. Proc. Natl. Acad. Sci. USA.

[17] Vengust M., Jager M.C., Zalig V., Cociancich V., Laverack M., Renshaw R.W., Dubovi E., Tomlinson J.E., Van de Walle G.R., Divers T.J. (2020). First report of equine parvovirus-hepatitis-associated Theiler’s disease in Europe. Equine Vet. J..

[18] Badenhorst M., de Heus P., Auer A., Tegtmeyer B., Stang A., Dimmel K., Tichy A., Kubacki J., Bachofen C., Steinmann E. (2021). Active equine parvovirus-hepatitis infection is most frequently detected in Austrian horses of advanced age. Equine Vet. J..

[19] Lu G., Sun L., Ou J., Xu H., Wu L., Li S. (2018). Identification and genetic characterization of a novel parvovirus associated with serum hepatitis in horses in China. Emerg. Microbes Infect..

[20] Lu G., Wu L., Ou J., Li S. (2020). Equine Parvovirus-Hepatitis in China: Characterization of Its Genetic Diversity and Evidence for Natural Recombination Events Between the Chinese and American Strains. Front. Vet. Sci..

[21] de Moraes M.V.D.S., Salgado C.R.S., Godoi T.L.O.S., de Almeida F.Q., Chalhoub F.L.L., de Filippis A.M.B., de Souza A.M., de Oliveira J.M., Figueiredo A.S. (2021). Equine parvovirus-hepatitis is detected in South America, Brazil. Transbound Emerg. Dis..

[22] Ramsauer A.S., Badenhorst M., Cavalleri J.V. (2021). Equine parvovirus hepatitis. Equine Vet. J..

[23] Horse Registry The current Status of Registered Horses in South Korea. https://www.horsepia.com/pa/hh/PAHH3000/pHorseRegStateMain.do.

[24] Sergeant E.S.G. Epitools Epidemiological Calculators. http://epitools.ausvet.com.au.

[25] Kumar S., Stecher G., Tamura K. (2016). MEGA7: Molecular Evolutionary Genetics Analysis Version 7.0 for Bigger Datasets. Mol. Biol. Evol..

[26] Waddell P.J., Steel M.A. (1997). General Time-Reversible Distances with Unequal Rates across Sites: Mixing Γ and Inverse Gaussian Distributions with Invariant Sites. Mol. Phylogenet. Evol..

[27] Orsini J.A., Divers T.J. (2008). Equine Emergencies.

[28] Tomlinson J.E., Van de Walle G.R., Divers T.J. (2019). What Do We Know About Hepatitis Viruses in Horses?. Vet. Clin. N. Am. Equine Pract..

[29] Kopper J.J., Schott H.C., Divers T.J., Mullaney T., Huang L., Noland E., Smedley R. (2020). Theiler’s disease associated with administration of tetanus antitoxin contaminated with nonprimate (equine) hepacivirus and equine parvovirus-hepatitis virus. Equine Vet. Educ..

[30] Animal Disease Control Division The Current List of Registered Animal Medicine (3rd Quarter of 2021). http://www.qia.go.kr/viewwebQiaCom.do?id=53739&type=2_23lylyy.

[31] Tomlinson J.E., Jager M., Struzyna A., Laverack M., Fortier L.A., Dubovi E., Foil L.D., Burbelo P.D., Divers T.J., Van de Walle G.R. (2020). Tropism, pathology, and transmission of equine parvovirus-hepatitis. Emerg. Microbes Infect..

