# Supplementary material for: Molecular Prevalence of Equine Parvovirus-Hepatitis in the Sera of Clinically Healthy Horses in South Korea
Source: Vet Sci. 2021 Nov 19;8(11):282. doi: 10.3390/vetsci8110282 (PMC8619122; doi:10.3390/vetsci8110282)
Supplement: Supplementary file 1 [file vetsci-08-00282-s001.zip › vetsci-1443023-SI.pdf]

veterinary sciences

MDPI

## Supporting Material

# Molecular Prevalence of Equine Parvovirus-Hepatitis in the Sera of Clinically Healthy Horses in South Korea

**Sang-Kyu Lee [1], Dongsun Park [2] and Inhyung Lee [3,*]**

**Table S1.** Detailed race results of Thoroughbred race horses used for the anlalysis of the relevance between performance levels and EqPV-H infection.

| Sample | KRA property | Breed | Purpose | Age | Race day (year-month-day) | Rank | Number of participants | Race rank percentage[1] | Performance level | EqPV-H infection |
|---|---|---|---|---|---|---|---|---|---|---|
| KRA57 | KRA Seoul | Thoroughbred | Racing | 5 | 2021-05-16 | 11 | 11 | 100.0% | Low level | positive |
| KRA58 | KRA Seoul | Thoroughbred | Racing | 4 | 2021-05-16 | 2 | 11 | 18.2% | High level | positive |
| KRA60 | KRA Seoul | Thoroughbred | Racing | 4 | 2021-05-16 | 7 | 11 | 63.6% | Moderate level | positive |
| KRA62 | KRA Seoul | Thoroughbred | Racing | 4 | 2021-05-16 | 12 | 12 | 100.0% | Low level | positive |
| KRA66 | KRA Seoul | Thoroughbred | Racing | 5 | 2021-05-16 | 6 | 9 | 66.7% | Moderate level | positive |
| KRA68 | KRA Seoul | Thoroughbred | Racing | 4 | 2021-05-16 | 2 | 9 | 22.2% | High level | positive |
| KRA106 | KRA Seoul | Thoroughbred | Racing | 5 | 2021-05-22 | 10 | 12 | 83.3% | Low level | positive |
| KRA129 | KRA Seoul | Thoroughbred | Racing | 5 | 2021-05-23 | 8 | 11 | 72.7% | Low level | positive |
| KRA151 | KRA Seoul | Thoroughbred | Racing | 7 | 2021-05-23 | 10 | 14 | 71.4% | Low level | positive |
| KRA270 | KRA Busan | Thoroughbred | Racing | 4 | 2021-06-12 | 6 | 10 | 60.0% | Moderate level | positive |
| KRA289 | KRA Busan | Thoroughbred | Racing | 4 | 2021-06-13 | 7 | 14 | 50.0% | Moderate level | positive |

| | | | | | | | | | | |
|---|---|---|---|---|---|---|---|---|---|---|
| KRA14 | KRA Seoul | Thoroughbred | Racing | 5 | 2021-05-16 | 4 | 12 | 33.3% | High level | negative |
| KRA47 | KRA Seoul | Thoroughbred | Racing | 4 | 2021-05-16 | 3 | 13 | 23.1% | High level | negative |
| KRA49 | KRA Seoul | Thoroughbred | Racing | 5 | 2021-05-16 | 9 | 13 | 69.2% | Low level | negative |
| KRA61 | KRA Seoul | Thoroughbred | Racing | 5 | 2021-05-16 | 8 | 11 | 72.7% | Low level | negative |
| KRA64 | KRA Seoul | Thoroughbred | Racing | 4 | 2021-05-16 | 1 | 9 | 11.1% | High level | negative |
| KRA71 | KRA Seoul | Thoroughbred | Racing | 6 | 2021-05-16 | 7 | 11 | 63.6% | Moderate level | negative |
| KRA105 | KRA Seoul | Thoroughbred | Racing | 3 | 2021-05-22 | 4 | 13 | 30.8% | High level | negative |
| KRA110 | KRA Seoul | Thoroughbred | Racing | 4 | 2021-05-23 | 3 | 12 | 25.0% | High level | negative |
| KRA140 | KRA Seoul | Thoroughbred | Racing | 6 | 2021-05-23 | 2 | 11 | 18.2% | High level | negative |
| KRA246 | KRA Busan | Thoroughbred | Racing | 3 | 2021-06-12 | 7 | 14 | 50.0% | Moderate level | negative |
| KRA277 | KRA Busan | Thoroughbred | Racing | 5 | 2021-06-13 | 3 | 14 | 21.4% | High level | negative |

[1] The percentage of rank divided by the number of participating animals in the race.
